# Comparing the Lecture-Based Learning With the Four-Component Instructional Design (4C/ID) Model of Learning in Enhancing the Skills of Consent-Taking in the Emergency Department: A Quasi-experimental Study

**DOI:** 10.7759/cureus.94133

**Published:** 2025-10-08

**Authors:** Abdus Salam Khan, Muhammad Nasir Ayub Khan, Muhammad I Khan

**Affiliations:** 1 Emergency Medicine, Shifa International Hospitals, Islamabad, PAK; 2 Emergency Medicine, Shifa Tameer-E-Millat University, Islamabad, PAK; 3 Anesthesia and Critical Care, Shifa International Hospitals, Islamabad, PAK; 4 General Surgery, Shifa Tameer-E-Millat University, Islamabad, PAK

**Keywords:** 4c/id model, emergency medicine, informed consent, interprofessional education, simulation training

## Abstract

Introduction

The emergency department (ED) is the first point of care for critically ill patients who require rapid stabilization and treatment, which frequently involves obtaining informed consent. The environment of the ED is challenging due to the complex interaction between staff and patients and their families, with numerous tasks needing to be performed within a limited time window. That is why consent-taking in the ED by healthcare professionals and nurses is sometimes inconsistent with best practices, leading to ethical and legal concerns. Informed consent-taking in the ED is critical yet challenging, with traditional lecture-based training often failing to equip healthcare professionals with practical skills. This study compares the Four-Component Instructional Design (4C/ID) model, a task-driven, interactive approach with lecture-based learning (traditional methods) for enhancing consent-taking skills.

Methods

A quasi-experimental study was conducted with 21 emergency care professionals, randomized into two groups: the control group underwent traditional lecture-based training, and the intervention group received 4C/ID-based training (simulations and role-playing). Outcomes were assessed via pre- and post-tests (knowledge retention), Objective Structured Clinical Examinations (OSCEs; practical skills), and focus group discussions (qualitative feedback on comfort/confidence).

Results

Both the control and intervention groups showed similar knowledge retention, but the 4C/ID group demonstrated better skill performance in OSCEs than traditional training. Qualitative analysis revealed that the 4C/ID participants reported greater confidence, understanding, and satisfaction with the training.

Conclusion

The 4C/ID model was shown to be promising in teaching consent-taking skills in the ED and increasing the healthcare professional’s confidence, compared to traditional lectures. This evidence supports adopting interactive, task-based training for ethical communication in high-pressure clinical settings like the ED.

## Introduction

Healthcare decision-making in emergency settings poses unique challenges. Healthcare staff must balance patients' autonomy with the urgent delivery of care [1]. In the emergency department (ED), this balance is often compromised. The time-sensitive and high-stress environment makes taking informed consent difficult [2-3], even though it is essential for patient-centered care and improved outcomes. Patients and families have frequently been found in states of anxiety, confusion, and emotional distress, further complicating communication and decision-making. In practice, consent-taking often falls short due to time constraints, complex decisions, and insufficient training [4]. Traditional didactic teaching is inadequate, as it does not provide the experiential preparation needed for real-world emergency scenarios [5].

Active learning strategies such as task-based learning and role-play are increasingly recognized as effective for skill development in healthcare education [5]. However, their systematic application in emergency care, particularly within structured frameworks like the Four-Component Instructional Design (4C/ID) model, remains underexplored [6]. Various studies described the value of instructional design, like the 4C/ID model, and showcase the value of teaching through role play [7-9]. While interprofessional learning has been shown to enhance collaboration, its integration with 4C/ID in high-pressure clinical settings has not been adequately studied. In Pakistan, consent practices are especially inconsistent, relying heavily on observational learning and occurring in a context where awareness of patient rights is limited [7].

This study evaluated the effectiveness of the 4C/ID model in improving informed consent skills among interprofessional ED staff. It also explored participants’ experiences and the model’s impact on interprofessional collaboration.

## Materials and methods

Study design

We conducted a quasi-experimental study to evaluate the effectiveness of a task-based learning intervention grounded in the 4C/ID model compared to a lecture-based study [9]. We hypothesized that the 4C/ID model is more effective than lecture-based learning for teaching communication skills in the ED. The intervention targeted four key elements of informed consent: capacity, communication, voluntary decision-making, and disclosure. The primary outcome of the study was the Objective Structured Clinical Examination (OSCE) score, assessed immediately after the intervention, with multiple-choice question (MCQ) scores as a secondary endpoint.

The effectiveness of the training was assessed using Kirkpatrick’s four-level evaluation model [10] with Level 1 (Reaction): Participant perceptions were explored through a structured focus group discussion; Level 2 (Learning): Knowledge was measured using pre- and post-intervention multiple-choice tests; and Levels 3 and 4 (Behavior and Results): Skills and application were assessed via OSCE stations and standardized simulated scenarios.

Setting and participants

The study was conducted in the ED of Shifa International Hospital, a tertiary care teaching facility in Islamabad, Pakistan. Eligible participants were emergency physicians and nurses with at least one year of clinical experience. Exclusion criteria included prior participation in 4C/ID-based training or documented communication impairments. Recruitment was conducted via departmental WhatsApp groups and direct invitations. Written informed consent was obtained from all participants (Appendix A).

Sampling and randomization

A total of 21 healthcare professionals (14 physicians and seven nurses) participated in the study. To ensure balance by gender and professional role, participants were stratified into four groups (male nurse, female nurse, male doctor, female doctor) and then randomly allocated into one of the two study groups using a lottery method. Since the staff comprised young individuals both in doctors and nurses group, the vast majority had the same experience level. 

Interventions

Participants were allocated to one of two groups: the control group (Group A) received a one-hour didactic lecture on informed consent. The intervention group (Group B) attended a four-hour interactive workshop designed according to 4C/ID principles. This included a brief lecture, followed by progressively complex role-play scenarios simulating real consent-taking encounters (Appendix B). Facilitators provided structured feedback, gradually reducing support to encourage learner autonomy.

Data collection instruments

We used the following instruments to evaluate the three levels of Kirkpatrick: level 2 (knowledge acquired), and levels 3 and 4 (skill and behavior change). We used pre- and post-intervention knowledge assessments to measure participants knowledge acquisition, addressing research question through 25 MCQs about the training content (Appendix C). The MCQs were prepared by two educationalists, pilot-tested on non-participants, and reviewed by an expert panel before use in this study.

We evaluated skills and behavior (levels 3 and 4) assessment by OSCE stations (Appendix D). The content of the OSCEs were blueprint-aligned to the four domains of informed consent. Each station was assessed by two independent, trained raters using standardized checklists. Standardized patients were employed to ensure realism. The evaluators of the OSCE stations were blinded to the participant's group to minimize bias. These evaluators were junior faculty and not the participants of the study.

The qualitative component of data was collected from purposive sample of six intervention participants, representing variation in gender and role, who participated in a focus group discussion (Appendix E). Sessions were audio-recorded, transcribed verbatim, and thematically analyzed following Braun and Clarke’s framework [11]. Two researchers independently coded the data, resolving discrepancies through discussion to enhance credibility.

Data analysis

Data was entered and statistical analyses were conducted using IBM SPSS Statistics for Windows, Version 23 (Released 2015; IBM Corp., Armonk, New York, United States). Descriptive statistics (mean, standard deviation, and proportions) were used to summarize demographic characteristics such as age, gender, and professional distribution of participants. For inferential analysis, independent sample t-test was applied to compare the pre-test and post-test scores between Group A (control group) and Group B (intervention group), and also to assess the differences in OSCE scores between the two groups. To evaluate the range of the mean difference in scores and assess the precision of the results, 95% Confidence Interval (CI) was used and statistical significance was set at p<0.05. However, p-values greater than this threshold were also interpreted in the context of educational and practical relevance.

The focused group discussion (FGD) was thematically analyzed to identify participants perception of the teaching methods, with a particular focus on the 4C/ID model of teaching.

## Results

Participant characteristics

A total of 21 healthcare professionals participated in the study (14 physicians and nurse nurses; 12 female participants and nine male participants). Participants were randomized into Group A (traditional lecture, n=10) and Group B (4C/ID-based intervention, n=11). Demographic characteristics, including gender, profession, and mean age, were balanced across groups (Table 1).

**Table 1:** Demographics of study participants, by group

Characteristics	Group A (n=10)	Group B (n=11)	Total (n=21)
Gender			
Female	6	6	12
Male	4	5	9
Profession			
Doctors	7	7	14
Nurses	3	4	7
Mean age (years)	29.1 ± 3.2	26.4 ± 2.8	27.7 ± 3.2

Quantitative findings

All participants took part in the class and also the OSCE stations, and there were no dropouts. 

Knowledge Outcomes

Baseline pre-test scores showed no significant difference between groups (Group A: 21.00 ± 3.20 vs. Group B: 21.18 ± 2.60; p=0.887). Both groups improved after their respective interventions, but post-test scores remained comparable (Group A: 21.90 ± 2.38 vs. Group B: 21.73 ± 2.87; p=0.883).

Skills and Performance Outcomes

Group B achieved higher mean OSCE scores compared with Group A (18.64 ± 1.22 vs. 17.44 ± 2.46), although this difference was not statistically significant (p=0.168). The results failed to show any clear superiority or any potential benefit of the 4C/ID model for practical skill acquisition (Table 2).

**Table 2:** Test scores by group OSCE: Objective Structured Clinical Examination; *The value of statistical significance was p<0.05.

Test Score	Group A (Mean ± SD)	Group B (Mean ± SD)	p-value*
Pre-test	21.00 ± 3.20	21.18 ± 2.60	0.887
Post-test	21.90 ± 2.38	21.73 ± 2.87	0.883
OSCE	17.44 ± 2.46	18.64 ± 1.22	0.168

Experience of the participants in the ED ranged from one to six years (mean 2.19; SD 1.401). Due to a high turnover rate in the department and a training program for emergency medicine, the majority of participants had an experience of one or two years. Due to small sample size of our experiment, plotting the experience against the OSCE result showed no effect due to the years of ED experience (Table 3). 

**Table 3:** Adjusted post-intervention OSCE scores by teaching method after controlling for years of emergency department experience (ANCOVA) ^a^Computed using alpha=0.05; The F tests the effect of group. This test is based on the linearly independent pairwise comparisons among the estimated marginal means. OSCE: Objective Structured Clinical Examination; ANCOVA: Analysis of covariance

	Sum of squares	df	Mean square	F	Sig.	Partial Eta Squared	Noncentrality parameter	Observed power^a^
Contrast	6.331	1	6.331	1.654	0.215	0.084	1.654	0.23
Error	68.898	18	3.828					

Qualitative findings

Thematic analysis of the focus group discussions with intervention participants identified five major themes, which complemented the quantitative results and provided a deeper insight into the learning experience.

Challenges With Traditional Lectures

Participants described lecture-based sessions as information-dense, fast-paced, and difficult to retain, with limited opportunity for application.

“They give you too much information in a short span of time, so it’s hard to remember.” - Participant 3

Positive Perception of Interactive Learning

The 4C/ID-based sessions were valued for their engaging and participatory nature, which supported better retention and understanding.

 “I wish I could learn everything through these interactive sessions.” - Participant 6

Role-Play as a Transformative Tool

Role-play exercises were highlighted as particularly effective in clarifying concepts and building confidence in consent-taking.

“My concepts became clearer when I saw things playing out in front of my eyes.” - Participant 4

Interprofessional Collaboration

Learning in mixed groups of doctors and nurses promoted mutual respect, understanding of roles, and appreciation of teamwork.

“It helped me understand the role of doctors, which will likely assist me in future collaborations.” - Participant 3

Improved Team Culture and Perceived Value

Participants emphasized that the interprofessional, interactive environment fostered stronger team culture and enhanced the perceived value of training.

“These interactions helped me understand more and created an interprofessional environment that was more helpful than I had imagined.” - Participant 2

## Discussion

Informed consent is both a legal mandate and an ethical cornerstone of medical practice. Within the high-acuity environment of the ED, where time pressures and patient vulnerability are heightened, the process becomes even more complex. Equipping ED staff with the skills required to obtain valid consent is therefore critical. Traditional training methods have relied heavily on lectures, but recent advances in the education of health professionals increasingly emphasize experiential and interactive approaches designed to enhance skill transfer, interprofessional collaboration, and clinical preparedness.

This mixed-methods study compared conventional lecture-based teaching with a structured, task-based intervention grounded in the 4C/ID model. Quantitative findings revealed no significant differences in knowledge acquisition between groups, a pattern consistent with prior studies across medical education, where didactic and active approaches often achieve similar outcomes in theoretical learning. Freeman and colleagues, in their meta-analysis of STEM education, similarly found that factual knowledge can be conveyed effectively via both methods, although active strategies were associated with superior learner engagement and retention [6]. Our findings reinforce this distinction between short-term knowledge acquisition and long-term or applied learning benefits.

Although the difference in OSCE performance did not reach statistical significance, the intervention group consistently outperformed the control group, suggesting a trend toward enhanced practical skill development through the 4C/ID model. This aligns with prior evidence demonstrating that 4C/ID-based instruction facilitates the acquisition and transfer of complex professional skills. Comparable results have been observed in nursing and interprofessional training, where role-play improved learners’ confidence, communication, and ability to manage real-world interactions [12]. A meta-analysis done on the use of educational programs developed with 4C/ID showed a higher impact on performance [12]. Together, these results indicate that while lectures can disseminate core knowledge efficiently, experiential, scaffolded methods are better suited to fostering the nuanced communication and decision-making skills required for consent-taking [13].

The qualitative findings provide valuable depth to these observations. Participants in the lecture group acknowledged the efficiency of structured delivery but described difficulties with retention and application due to information overload, limitations well documented in the literature [14]. In contrast, those in the intervention group reported greater engagement, enjoyment, and retention. Role-play was perceived as transformative, enhancing both conceptual clarity and practical confidence. These reflections echo earlier studies of interprofessional simulation, which highlight improved teamwork, communication, and professional role understanding as key benefits of interactive approaches [15].

The interprofessional element of the intervention was particularly noteworthy. Nursing participants emphasized the value of training alongside physicians, fostering mutual respect and appreciation of roles. This finding is consistent with reports from diverse settings, including the Middle East, where interprofessional education has been associated with improved collaboration and safer patient care [16]. Within the ED context, where effective teamwork is essential for both patient safety and efficiency, the contribution of such approaches is particularly salient [17-18].

Taken together, these findings support the growing consensus that lectures remain effective for rapid dissemination of knowledge, but complex, communication-intensive skills such as consent-taking are best developed through structured, interactive, and interprofessional strategies [19-20]. The 4C/ID model, with its emphasis on authentic tasks, scaffolding, and guided practice, provides a particularly suitable framework for this type of training.

Practical implications

This study underscores the need for emergency medicine educators and clinical leaders to adopt a blended approach to training for consent-taking. Lectures should continue to be used for delivering foundational knowledge efficiently, but they must be complemented by structured 4C/ID-based workshops and role-play to ensure meaningful skill transfer, engagement, and learner confidence. EDs should actively integrate such short, focused sessions into routine staff development programs, as they can be delivered without disrupting clinical operations and have the potential to significantly strengthen communication competencies in time-sensitive environments. Moreover, designing consent-training initiatives for mixed groups of physicians and nurses should become standard practice, as this fosters interprofessional collaboration and mutual respect, ultimately contributing to safer patient care [21-22]. Given its scalability and minimal resource requirements, the 4C/ID model represents a practical and evidence-based solution that should be prioritized, particularly in low- and middle-income settings where simulation facilities and training resources are limited.

Limitations

This study has several limitations. The small sample size, single-center design, and short intervention duration restrict the generalizability of findings. The lack of long-term follow-up further limits conclusions regarding the sustainability of the observed improvements. Conducting the intervention in a busy ED, characterized by high staff turnover and scheduling constraints, also posed challenges to delivering consistent, high-quality teaching. Despite these limitations, the study offers valuable insights for educators working in resource-constrained environments and demonstrates the feasibility of implementing structured, task-based training within demanding ED settings.

## Conclusions

This study demonstrated that while both lecture-based and 4C/ID-based methods are effective for conveying theoretical knowledge, the 4C/ID model offers added advantages for practical skill development and interprofessional collaboration. Interactive, task-based approaches such as role-play appear particularly well-suited for preparing healthcare professionals to manage ethically complex and communication-intensive processes such as informed consent in the ED. Future research on role-play should include larger, interprofessional, multi-center cohorts with longitudinal follow-up to examine sustainability, transferability, and cost-effectiveness of such interventions.

## Figures and Tables

**Figure 1:**
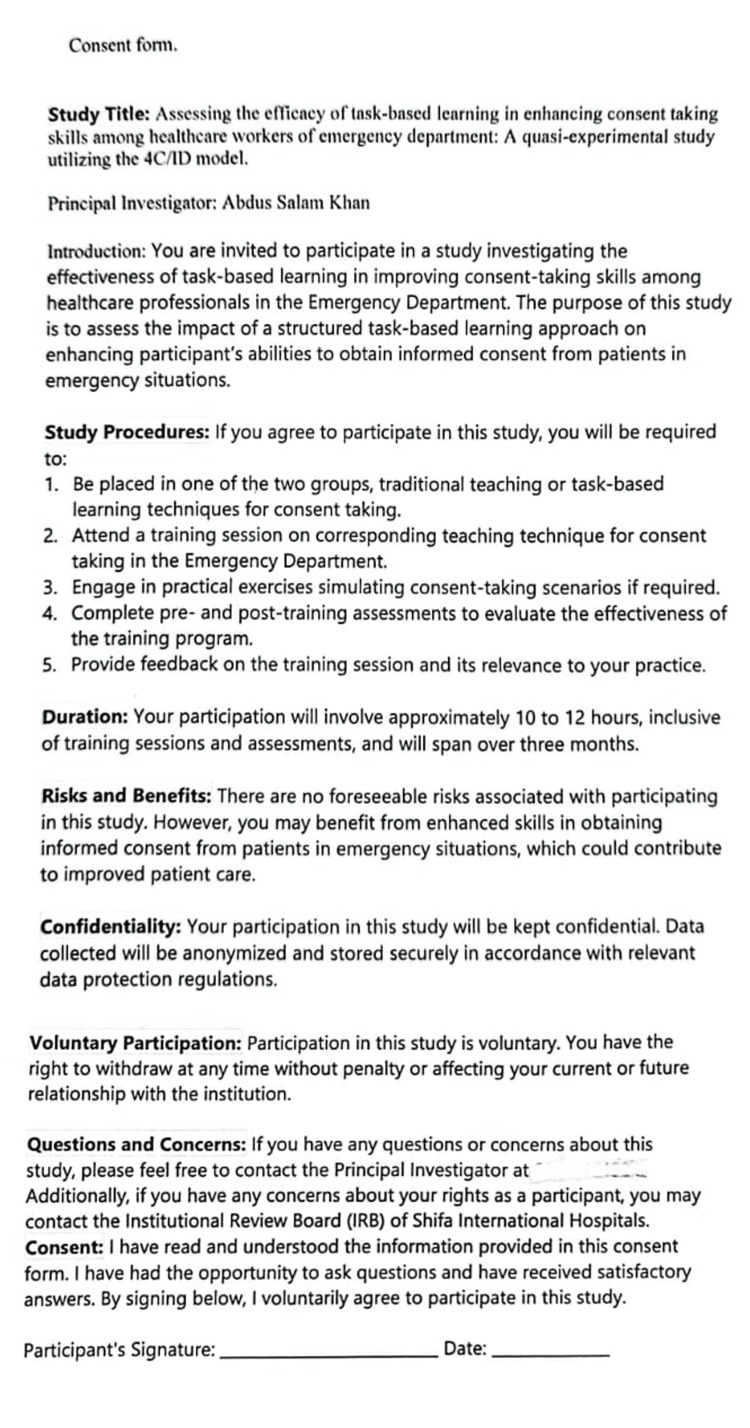
Consent form

## Appendices

Appendix A

Appendix B

Detailed Plan of the Course

1. Introduction and Overview (15 minutes)

Objective: Highlight the importance of informed consent in the emergency department.

Activities:

Brief presentation on ethical, legal, and practical implications of informed consent in the ED.

Open discussion to understand participants' baseline knowledge and experiences.

2. Foundation knowledge (30 minutes)

Objective: Teach the foundational principles of informed consent, including communication techniques and patient rights.

Activities:

Interactive lecture on key components of informed consent.

Discussion of challenges specific to ED settings (e.g., time constraints, emotional patients).

3. Learning tasks with role-playing (90 minutes)

Task 1 (30 minutes): Understanding key components

Objective: Break down the elements of effective consent-taking (e.g., providing information, ensuring understanding, decision-making capacity).

Method:

Divide participants into small groups (4 per group).

Each group identifies and discusses key components using a checklist.

Task 2 (30 minutes): Scenario-based role-playing

Objective: Apply the principles learned in a simulated consent-taking scenario.

Method:

Assign one participant per group as the "physician," one as the "patient," and others as observers.

Use case scenarios (e.g., informed consent for a lumbar puncture).

Rotate roles within the group.

Break (15 minutes)

4. Supportive information (30 minutes)

Objective: Teach skills such as active listening, addressing emotions, and explaining medical procedures effectively.

Method:

Case-based discussion highlighting communication strategies.

Examples of empathetic and patient-centered communication techniques.

5. Just-in-time information (30 minutes)

Objective: Provide specific, task-focused guidance.

Method:

Short videos showing ideal consent-taking practices.

Discussion of dos and don’ts in consent-taking.

6. Part-task practice (30 minutes)

Objective: Focus on specific consent-taking elements like explaining risks or checking patient understanding.

Method:

Role-play specific tasks in pairs.

Each task is supervised by an instructor, who provides immediate feedback.

7. Feedback and reflection (30 minutes)

Objective: Consolidate learning through feedback and self-reflection.

Method:

Peer feedback within groups based on performance checklists.

Instructors provide individualized feedback on strengths and areas for improvement.

Group reflection on challenges and lessons learned.

Resources needed:

Role-play scenarios (case studies).

Checklists for consent-taking components.

Videos of model performances.

Facilitators/instructors for supervision and feedback.

Feedback forms for peer and instructor evaluations.

Expected outcomes:

Improved knowledge of informed consent principles.

Enhanced communication and consent-taking skills.

Higher confidence and comfort in obtaining informed consent in ED settings.

Positive participant feedback regarding 4C/ID-based teaching.

Let me know if you'd like further customization or adjustments!

Appendix C

Pre- and Post-Test Questions

Consent-taking skills: Below is a list of 25 multiple-choice questions designed to assess knowledge, skills, and scenario-based understanding of consent-taking.

Which of the following is a key component of informed consent? a) Patient’s signature b) Disclosure of risks, benefits, and alternatives c) Verbal agreement by the patient d) Witness signature

Answer: b

Informed consent is primarily grounded in which ethical principle? a) Autonomy b) Beneficence c) Non-maleficence d) Justice

Answer: a

Which of the following patients has the capacity to provide informed consent? a) A 65-year-old with advanced dementia b) A 28-year-old with a mild fever and no cognitive issues c) A 40-year-old in shock and unable to speak coherently d) A 16-year-old with no parent or guardian present

Answer: b

When is implied consent typically applied in the Emergency Department? a) For elective procedures b) In life-threatening situations requiring immediate action c) When the patient refuses to sign a form d) For procedures requiring minimal risk

Answer: b

Which of the following is NOT an essential step in obtaining informed consent? a) Assessing the patient’s understanding b) Sharing risks and alternatives c) Documenting the patient’s decision d) Providing detailed medical terminology

Answer: d

Who is responsible for obtaining informed consent for a procedure in the ED? a) The nurse on duty b) The doctor performing the procedure c) The hospital administrator d) Any healthcare worker available

Answer: b

Informed consent must be: (Select the best answer) a) Voluntary, informed, and documented b) Comprehensive, signed, and notarized c) Verbal, witnessed, and stored electronically d) Short, simple, and physician-centric

Answer: a

What should you do if a patient refuses treatment after being fully informed of the risks? a) Proceed with the treatment for their safety b) Document the refusal and respect their decision c) Call a family member to override the decision d) Avoid documenting the refusal to prevent legal issues

Answer: b

Which of the following is most likely to undermine the consent-taking process? a) Using plain language b) Rushing the explanation due to time constraints c) Checking the patient’s understanding d) Allowing the patient to ask questions

Answer: b

What is the primary goal of obtaining informed consent? a) Protecting the hospital from lawsuits b) Allowing patients to make an informed decision c) Following legal documentation protocols d) Ensuring all risks are disclosed

Answer: b

Which communication skill is most important when explaining a procedure to a patient? a) Speaking in medical jargon b) Maintaining eye contact and listening actively c) Giving the patient minimal time to respond d) Using diagrams without further explanation

Answer: b

Role-playing in medical education is beneficial for: a) Practicing theoretical knowledge b) Developing patient-centered communication skills c) Memorizing facts about medical procedures d) Observing real patient interactions

Answer: b

How should you respond if a patient appears confused during the consent discussion? a) Move forward with the consent process b) Re-explain the information in simpler terms c) Ask another healthcare worker to take over d) Focus on documenting the consent process

Answer: b

Which statement best demonstrates empathy during consent-taking? a) “I understand this is overwhelming, and I am here to answer your questions.” b) “This is a standard procedure, so you have nothing to worry about.” c) “The decision is yours, but I recommend you agree.” d) “I’ve explained everything, so let’s proceed.”

Answer: a

In role-playing exercises, the primary focus should be on: a) Memorizing scripts b) Practicing communication and decision-making skills c) Perfecting clinical procedures d) Winning peer approval

Answer: b

What should you do if a simulated patient refuses consent during role-playing? a) Force them to agree for the exercise to continue b) Explore their concerns and address their questions c) End the exercise immediately d) Ignore the refusal and proceed

Answer: b

What is a key outcome of using role-playing in consent-taking training? a) Better theoretical knowledge retention b) Enhanced confidence and practical communication skills c) Avoidance of real-life patient interactions d) Development of improvisational techniques

Answer: b

Which of the following improves consent-taking communication? a) Interrupting the patient to clarify details b) Allowing the patient to express concerns and ask questions c) Using complex language to appear knowledgeable d) Focusing solely on obtaining a signature

Answer: b

In simulated role-playing, the observer’s role is to: a) Criticize the participant’s performance b) Provide constructive feedback based on the scenario c) Take notes without providing feedback d) Grade the participant for the facilitator

Answer: b

Role-playing is particularly effective for improving which aspect of consent-taking? a) Procedural knowledge b) Communication and empathy skills c) Knowledge of legal terms d) Clinical decision-making

Answer: b

Scenario: A patient with chest pain requires a procedure, but they seem distracted and anxious. What is the best initial approach? a) Provide the consent form and ask for a quick signature b) Ask if they have any concerns and explain in clear, simple terms c) Proceed with the procedure without discussing consent d) Tell them they must listen carefully

Answer: b

Scenario: A patient’s spouse insists on making decisions for the patient, even though the patient is capable of deciding. What should you do? a) Respect the spouse’s request b) Focus on the patient’s autonomy and obtain consent from them c) Ask another staff member to mediate d) Proceed without discussing the matter

Answer: b

Scenario: During consent-taking, the patient asks about alternative treatments you are unfamiliar with. What should you do? a) Avoid addressing the question to stay on track b) Inform them you will consult a colleague and provide accurate information c) Discourage them from considering alternatives d) Assume the alternative is not relevant

Answer: b

Scenario: A patient refuses consent for a non-urgent procedure after understanding the risks and benefits. What is your next step? a) Respect their decision and document the refusal b) Try to persuade them by emphasizing the risks c) Proceed without their consent d) Ask a colleague to take over

Answer: a

Scenario: A simulated patient keeps interrupting the consent discussion. How should you handle it? a) Politely acknowledge their concerns and redirect the conversation b) Ignore the interruptions and continue c) End the discussion d) Ask another staff member to intervene

Answer: a

Appendix D

Skill Testing Stations for Consent-Taking Training

These 12 stations are designed to evaluate trainees' practical skills, communication abilities, and understanding of consent-taking in emergency settings. Each station will test different aspects of the process. Simulated patients (SPs) and/or observers will be used to assess performance.

Checklists for skill testing stations: Each station has a detailed checklist to ensure standardized evaluation. Observers will score each criterion on a scale of 1-5 (1 = Poor, 5 = Excellent).

Implementation notes:

Time per station: 10 minutes (8 minutes for the task, 2 minutes for transition/feedback)

Observers/Assessors: Each station requires an observer to assess performance using a structured checklist.

Scoring: A rubric with specific criteria for each station will be used to ensure consistency and fairness.

Scoring guidelines: 1-2: Poor performance; significant gaps; 3: Satisfactory; meets minimum expectations; 4: Good; above average with minor areas for improvement; 5: Excellent; meets all criteria to the highest standard

Let me know if you need additional customizations or specific rubrics for certain stations!

Station 1: Introduction and rapport building

Objective: Assess the trainee’s ability to initiate a conversation and establish rapport with the patient.

Scenario: You are meeting a patient for the first time who requires a minor surgical procedure. Begin the consent discussion.

Assessment criteria:

Introduces themselves professionally

Explains their role clearly

Demonstrates empathy and active listening

Station 1: Introduction and rapport building

Criteria 

Score (1-5)

Introduces themselves professionally

Clearly explains their role

Demonstrates empathy and active listening

Maintains appropriate body language

Engages patient to build trust and rapport

Total Score:     /25

Station 2: Explaining the procedure

Objective: Evaluate how effectively the trainee explains the procedure in simple terms.

Scenario: The patient requires a chest tube insertion. Explain what the procedure involves.

Assessment criteria:

Provides clear, concise, and jargon-free explanation

Includes key steps of the procedure

Checks for patient's understanding

Station 2: Explaining the procedure

Criteria

Score (1-5)

Provides a clear and concise explanation

Avoids medical jargon

Includes all key steps of the procedure

Checks for patient's understanding

Uses visual aids or diagrams if needed

Total Score:     /25

Station 3: Risk and benefit disclosure

Objective: Test the trainee’s ability to disclose risks and benefits accurately.

Scenario: A patient requires an urgent lumbar puncture. Explain the risks, benefits, and alternatives.

Assessment criteria:

Provides balanced information about risks and benefits

Mentions alternative options if available

Assesses patient comprehension

Station 3: Risk and benefit disclosure

Criteria

Score (1-5)

Clearly communicates risks and benefits

Balances the discussion without bias

Mentions alternative options

Encourages patient to ask questions

Confirms patient’s understanding

Total Score:      /25

Station 4: Assessing decision-making capacity

Objective: Assess the trainee’s ability to evaluate the patient’s capacity to provide informed consent.

Scenario: A 75-year-old patient with mild confusion needs a CT scan with contrast. Determine if they can consent.

Assessment criteria:

Asks relevant questions to assess understanding

Evaluates orientation, comprehension, and judgment

Documents findings appropriately

Station 4: Assessing decision-making capacity

Criteria

Score (1-5)

Asks relevant questions to assess understanding

Evaluates orientation, comprehension, and judgment

Considers emotional and cognitive factors

Documents findings appropriately

Demonstrates respect and professionalism

Total Score:       /25

Station 5: Handling patient anxiety

Objective: Test the trainee’s communication skills in calming an anxious patient.

Scenario: A young patient is visibly anxious about a central line insertion. Reassure them and proceed with the consent process.

Assessment criteria:

Acknowledges the patient’s concerns

Uses empathetic and calming language

Maintains a patient-centered approach

Station 5: Handling patient anxiety

Criteria

Score (1-5)

Acknowledges the patient’s concerns

Uses empathetic and calming language

Provides reassurance about the procedure

Encourages questions to address fears

Maintains a patient-centered approach

Total Score:      /25

Station 6: Dealing with language barriers

Objective: Assess how the trainee manages communication challenges.

Scenario: A non-English-speaking patient requires an emergency blood transfusion. Use an interpreter (role-played by an SP or observer) to obtain consent.

Assessment criteria:

Effectively uses the interpreter

Maintains clear and respectful communication

Ensures patient understanding

Station 6: Dealing with language barriers

Criteria

Score (1-5)

Effectively uses the interpreter

Maintains clear and respectful communication

Ensures patient understanding through feedback

Uses simple language and avoids jargon

Demonstrates cultural sensitivity

Total Score:       /25

Station 7: Managing family dynamics

Objective: Test how the trainee navigates family involvement in the consent process.

Scenario: The family of a conscious, competent patient insists on making decisions for them. Resolve the situation.

Assessment criteria:

Upholds the patient’s autonomy

Handles family concerns respectfully

Communicates effectively with all parties

Station 7: Managing family dynamics

Criteria

Score (1-5)

Upholds the patient’s autonomy

Handles family concerns respectfully

Communicates effectively with all parties

Balances family involvement appropriately

Maintains professionalism

Total Score:      /25

Station 8: Explaining alternatives

Objective: Assess the trainee’s ability to discuss alternative treatments.

Scenario: A patient requires wound debridement but is hesitant. Explain other treatment options.

Assessment criteria:

Clearly outlines alternative treatments

Discusses the pros and cons of each option

Encourages patient participation in decision-making

Station 8: Explaining alternatives

Criteria

Score (1-5)

Outlines alternative treatments clearly

Discusses pros and cons of each option

Encourages patient to participate in decision-making

Ensures patient understanding

Respects the patient’s preferences

Total Score:    /25

Station 9: Obtaining consent in a time-sensitive scenario

Objective: Evaluate how the trainee balances urgency and informed consent.

Scenario: A patient with a suspected ruptured spleen requires immediate surgery. Obtain consent while addressing the urgency.

Assessment criteria:

Explains the urgency appropriately

Provides essential information without overwhelming the patient

Maintains ethical standards

Station 9: Obtaining consent in a time-sensitive scenario

Criteria

Score (1-5)

Explains urgency clearly

Provides essential information concisely

Ensures patient understanding despite time constraints

Maintains ethical standards

Balances urgency with thoroughness

Total Score:   /25

Station 10: Documenting consent

Objective: Test the trainee’s ability to document the consent process.

Scenario: After obtaining verbal consent for a procedure, document it in the patient’s chart.

Assessment criteria:

Clearly and accurately documents the discussion

Includes all essential components (risks, benefits, patient understanding)

Uses proper medical terminology

Station 10: Documenting consent

Criteria

Score (1-5)

Accurately documents the discussion

Includes all essential components (risks, benefits, patient understanding)

Uses proper medical terminology

Highlights patient’s specific concerns

Documents patient’s questions and responses

Total Score:   /25

Appendix E

Conducting a Focused Group Interview for Teaching Assessment: Guidelines for Conducting Focused Group Discussion

1. Purpose of the Focused Group Interview

Objective: To gather qualitative feedback on the effectiveness of the 4C/ID-based teaching intervention on consent-taking skills.

Focus areas:

Knowledge acquisition

Skill improvement

Comfort level with the teaching method

Suggestions for improvement

2. Group size and composition

Ideal number of participants: 6-8 participants per group. This size is manageable for effective discussion while allowing for diverse opinions.

3. Preparing for the focused group

Facilitator: Assign a skilled moderator to guide the discussion neutrally.

Environment: Conduct the interview in a quiet, neutral setting to encourage open and honest responses.

Duration: Plan for 60-90 minutes per session.

Recording and notes: 

Use audio or video recording (with consent) to ensure no details are missed.

Have a notetaker for backup documentation.

4. Designing the discussion guide

Opening questions (Warm-up):

1. How did you feel about the teaching sessions on consent-taking?

2. What were your expectations before the training began?

Core questions:

Effectiveness of the 4C/ID-based teaching: 3. How effective was the training in improving your knowledge of consent-taking? 

4. Did you feel the 4C/ID model provided better clarity compared to traditional methods? Why or why not?

Skill application: 5. How confident are you in applying what you learned to real-life scenarios?

6. How did role-playing help you improve your communication and consent-taking skills?

Engagement and comfort: 7. Did you find the teaching methodology engaging? 

8. Was the training structured in a way that reduced your cognitive load (e.g., breaking tasks into simpler components)?

Comparison to traditional methods: 9. How does this method compare to other training sessions you’ve attended?

10. What specific aspects of this training stood out as effective or ineffective?

Feedback and suggestions: 11. What would you suggest to improve this training program? 

12. Were there any challenges or barriers you faced during the training?

5. Conducting the interview

Start by introducing the purpose of the session and setting ground rules (e.g., respect, confidentiality).

Begin with open-ended questions and follow up with probing questions to encourage deeper discussion.

Maintain a neutral tone to avoid influencing responses.

6. Data analysis

Transcription: Transcribe the recordings for analysis.

Thematic analysis: Identify recurring themes, trends, and insights from participants' responses.

Comparison: Compare feedback between intervention and control groups (if applicable).

7. Reporting results

Highlight common themes, unique perspectives, and actionable suggestions.

Categorize feedback into strengths, weaknesses, and areas for improvement.
